# The emerging regulatory roles of non-coding RNA in Ventilator-induced Lung Injury

**DOI:** 10.1016/j.ncrna.2026.01.001

**Published:** 2026-01-24

**Authors:** Qianqi Xiong, Ziyang Zhang, Yiping Bai, Li Liu, Yue Zhang, Jing Jia, Yingying Zhang

**Affiliations:** a Department of Anesthesiology, The Affiliated Hospital, Southwest Medical University, Luzhou, PR China; b Anesthesiology and Critical Care Medicine Key Laboratory of Luzhou, Southwest Medical University, Luzhou, PR China; c Department of Pain Management, The Affiliated Hospital, Southwest Medical University, Luzhou, PR China; d Academic Affairs Department, Clinical Medical College, Southwest Medical University, Luzhou, PR China

**Keywords:** Non-coding RNA (ncRNA), microRNA (miRNA), Long non-coding RNA (lncRNA), Ventilator-induced lung injury (VILI), Lung development

## Abstract

Ventilator-induced lung injury (VILI) is characterized by pathological features such as infiltration of inflammatory cells, increased alveolar permeability, and changes in lung compliance. The underlying mechanisms responsible for these manifestations during mechanical ventilation remain unclear. Low tidal volume ventilation and positive end-expiratory pressure (PEEP) can mitigate pulmonary edema and improve lung compliance. However, these strategies do not fully prevent VILI, and mortality remains high. Non-coding RNAs (ncRNAs) are involved in mechanotransduction processes during ventilation by modulating cellular functions through the activation of inflammatory signaling pathways, such as Toll-like receptor (TLR), Janus kinase/signal transducer and activator of transcription (JAK-STAT), and nuclear factor kappa-B (NF-κB). These pathways contribute to the development of VILI and possess notable diagnostic, differential diagnostic, and therapeutic potential. This review offers a comprehensive evaluation of current research on microRNAs and long non-coding RNAs in relevant models of VILI.

## 1 Introduction

The Ventilator-induced lung injury (VILI) results from factors associated with mechanical ventilation, such as alveolar overdistension and the cyclical opening and closing of alveoli. Additionally, reductions in surfactant content and local hypoxia contribute to tissue damage. These factors can directly or indirectly stimulate the release of cellular mediators, facilitating their dissemination and colonization [1]. Characteristic pathological features of VILI usually manifest as alveolar collapse, hyaline membrane formation, increased vascular permeability, and pulmonary edema [2,3]. Some critical pathogenic mechanisms of VILI, such as pulmonary inflammatory cell infiltration and highly induced cytokine expression, have been further understood in recent years [1]. However, our knowledge regarding the underlying initiating and regulatory pathways that govern these inflammatory responses remains limited. The prevailing viewpoint in the scientific community is that the occurrence of VILI is closely associated with multiple factors. Specifically, these factors include the release of pro-inflammatory cytokines, production of reactive oxygen species (ROS), activation of the complement system and mechanical signal transduction. These factors are widely recognized as central drivers of VILI pathogenesis. [4]. These factors are widely recognized as central drivers of VILI pathogenesis.

Non-coding RNA (ncRNA) is a class of RNA molecules that do not encode proteins but are essential in regulating gene expression, cellular growth, and disease development [5,6]. These ncRNAs are transcribed from the human genome [7]. Based on size, ncRNAs can be categorized into two groups: small (less than 200 nucleotides) and long (more than 200 nucleotides) ncRNAs. In terms of their functional roles, ncRNAs can be further classified into regulating ncRNAs, i.e., lncRNAs, small interfering RNAs (siRNAs), piwi interacting RNAs (piRNAs), and microRNAs (miRNAs), and housekeeping ncRNAs, such as rRNAs, snRNAs, snoRNAs and tRNAs [8]. NcRNAs can activate inflammatory signaling pathways such as Toll-like receptors (TLR), Janus kinase/signal transducer and activator of transcription (JAK/STAT) and nuclear factor kappa-B (NF-κB), thus contributing to the development of VILI [9,10]. Increasing evidence has confirmed the potential of ncRNAs in diagnosing disease and treatment [9,11]. This review aims to summaries the regulatory functions of ncRNAs in lung development, pathological changes, and the pathogenesis of VILI, thereby providing valuable insights into the pathogenesis of VILI and identifying potential therapeutic targets.

## 2 NcRNAs participate in lung development

**Fig. 1:**
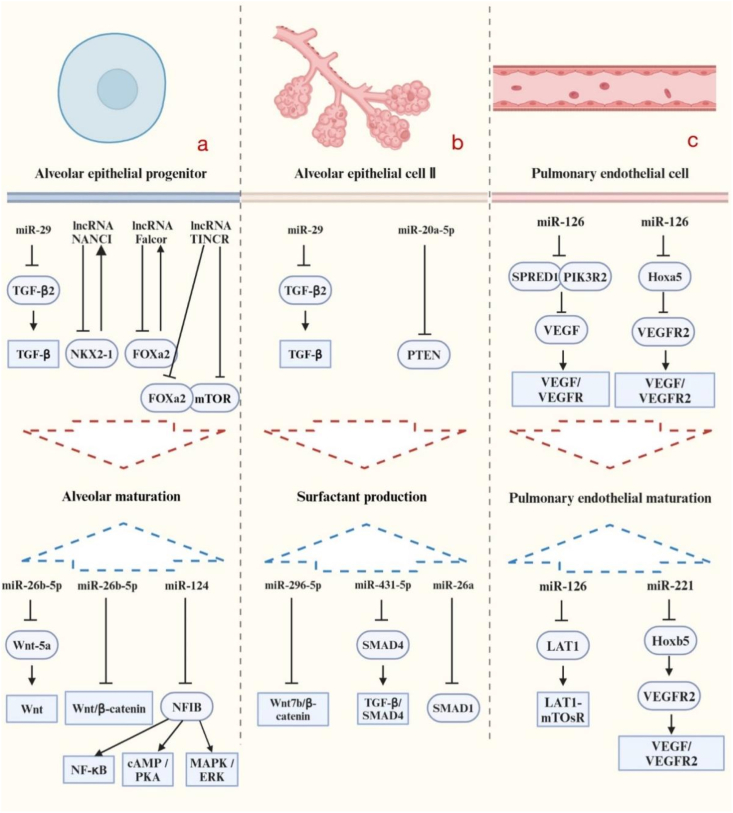
NcRNAs participate in lung development process. **a:** Affecting alveolar maturation; **b:** modulating surfactant production; **c:** regulating pulmonary endothelial maturation.

The lungs are subject to the coordinated control of various genes, which regulate each other in both spatial and temporal dimensions (Fig. 1). The lung exhibits a diverse expression profile of ncRNAs. Researchers have found that signaling pathways involved in normal lung physiological processes can influence lung development by modulating the stability and activity of ncRNA processing complexes [12]. MiRNAs possess complementary sequences to messenger RNAs (mRNAs) and have the ability to decrease mRNA expression [13]. The functionality of lncRNAs is linked with their subcellular localization. In the nucleus, they govern gene expression at the epigenetic and transcriptional levels, while in the cytoplasm, they regulate gene expression at the post-transcriptional and translational levels [14,15]. Table 1 provides a compilation of recently reported ncRNAs that participate in lung development.

**Table 1:** Overview of ncRNAs Modulating AEC Differentiation, Maturation, and Angiogenesis During lung development.

Roles	ncRNAs	Target	Pathway	References
Promoting AEC II differentiation	miR-29	TGF-β2	TGF-β signaling	[16]
Inhibiting AECII differentiation	miR-26b-5p	Wnt5a	Wnt signaling	[17]
	miR-615–3p	NA	Wnt/β-catenin	[18]
Inhibiting epithelial cell maturation	miR-124	NFIB	NF-κB, cAMP/PKA, and MAPK	[19]
Maintaining normal AEC differentiation	lncRNA NANCI	Nkx2-1	NANCI-Nkx2-1 feedback loop	[20]
Maintaining AEC epithelial homeostasis	lncRNA Falcor	Foxa2	Falcor - Foxa2 axis	[21]
Maintaining normal bronchial epithelial cell differentiation	lncRNA TINCR	SOX2 and NOTCH	NA	[22]
Promoting the production of pulmonary surfactant	miR-29	TGF-β2	TGF-β signaling	[16]
	miR-20a-5p	PTEN	NA	[23]
Inhibiting the production of pulmonary surfactant	miR-296–5p	Wnt7b/β-catenin	NA	[24]
	miR-26a	SMAD1	NA	[25,26]
	miR-431–5p	SMAD4	TGFβ/SMAD4	[27]
Promoting pulmonary macro angiogenesis	miR-126	SPRED1 and PIK3R2	VEGF/VEGFR	[28]
Inhibiting pulmonary micro angiogenesis	miR-126	LAT1	LAT1-mTOR	[29]
Inhibiting pulmonary angiogenesis	miR-221	Hoxb5	VEGF/VEGFR2	[30]
Promoting pulmonary angiogenesis	miR-130a	Hoxa5	VEGF/VEGFR2	[30]

Abbreviation: TGF-β2: Transforming growth factor β2; Wnt: Wingless-Type MMTV Integration Site Family; NFIB: nuclear factor I/B; cAMP: cyclic adenosine monophosphate; PKA: protein kinase A; MAPK: mitogen-activated protein kinase; NKX2-1: NK2 homeobox 1–expressing; NANCI: Nkx2-1-associated noncoding intergenic; Foxa2: Forkhead Box A2; TINCR: Terminal differentiation-induced non-coding RNA; SOX2: SRY-box transcription factor 2; PTEN: phosphatase and tensin homolog; SMAD: Drosophila mothers against decapentaplegic protein; SPRED1: sprouty related EVH1 domain containing 1; PIK3R2: phosphoinositide-3-kinase regulatory subunit 2; VEGF: vascular endothelial growth factor; LAT1: L-type amino acid transporter 1; LAT1-mTOR: L-type amino acid transporter 1-mammalian target of rapamycin; NA: not available.

### 2.1 NcRNAs in the differentiation of alveolar epithelial cells

NcRNAs play pivotal roles in alveolar epithelial cell (AEC) differentiation via several signaling pathways, including downregulating the transforming growth factor β (TGF-β) pathways [16], inhibiting Wnt5a and Wnt/β-catenin pathway [17,18], and inhibiting NF-κB, cAMP/PKA, and MAPK pathways [19]. Furthermore, lncRNAs drive lung epithelial differentiation by buffering transcription factor expression and regulating differentiation-related elements. For instance, lncRNA NANCI maintains AEC differentiation and alveolar structure through a feedback loop with the progenitor marker Nkx2-1 [20,31]. Similarly, lncRNA Falcor buffers FOXA2 to support epithelial regeneration and homeostasis by modulating SOX2 and NOTCH signaling components [21,22].

### 2.2 Roles of ncRNAs in pulmonary surfactant production

Pulmonary surfactant comprises complex lipoproteins produced by AEC II. However, elevated miR-296–5p levels in preterm infants could inhibit surfactant secretion via the Wnt7b/β-catenin pathway thus contributing to neonatal respiratory distress syndrome (NRDS) [24]. Similarly, miR-26a regulates surfactant synthesis in AEC II by targeting SMAD [25,26], while miR-29 and miR-20a-5p promote surfactant production by downregulating TGF-β2 and PTEN [16,23], respectively. Other miRNAs, such as miR-431–5p and miR-16, also modulate surfactant synthesis through pathways like TGF-β/SMAD4 or by enhancing SP-A expression [27,32]. Collectively, these findings demonstrate the critical regulatory role of miRNAs in pulmonary surfactant generation (Fig. 1b).

### 2.3 The functions of ncRNAs in pulmonary vascular growth

In both the pulmonary and systemic vasculatures, miR-126 plays a crucial role in regulating angiogenesis and maintaining vascular integrity [33]. It also exhibits tissue-specific functions. In large vessels, it enhances vascular endothelial growth factor (VEGF signaling by targeting SPRED1 and PIK3R2, promoting vascular integrity [28,34]. Conversely, in pulmonary microvessels, miR-126 inhibits the LAT1-mTOR axis, impairing endothelial proliferation [29,35]. Additionally, other miRNAs regulate pulmonary vascular formation with opposing effects: miR-221 targets Hoxb5 to downregulate VEGFR2 and inhibit angiogenesis [30,36], while miR-130a targets Hoxa5 to upregulate VEGFR2 and promote vascular expansion [30,37].

NcRNAs precisely orchestrate lung development by finely regulating gene expression across spatiotemporal dimensions. They are essential for alveolar epithelial cell differentiation, where lncRNAs (e.g., NANCI, Falcor) and miRNAs modulate critical signaling pathways, including TGF-β and Wnt/β-catenin, to maintain epithelial homeostasis. Regarding pulmonary surfactant production, specific miRNAs regulate synthesis and secretion via targets such as SMAD and PTEN, potentially influencing the susceptibility to neonatal respiratory distress syndrome. Moreover, ncRNAs control pulmonary vascular growth. Key regulators, such as miR-126, miR-221 and miR-130a, governs angiogenesis and vascular integrity through VEGF signaling mechanisms. Collectively, these diverse regulatory networks highlight the crucial role of ncRNAs in ensuring proper lung morphogenesis and physiological maturation.

## 3 NcRNAs participate in VILI via modulating the inflammation response

**Fig. 2:**
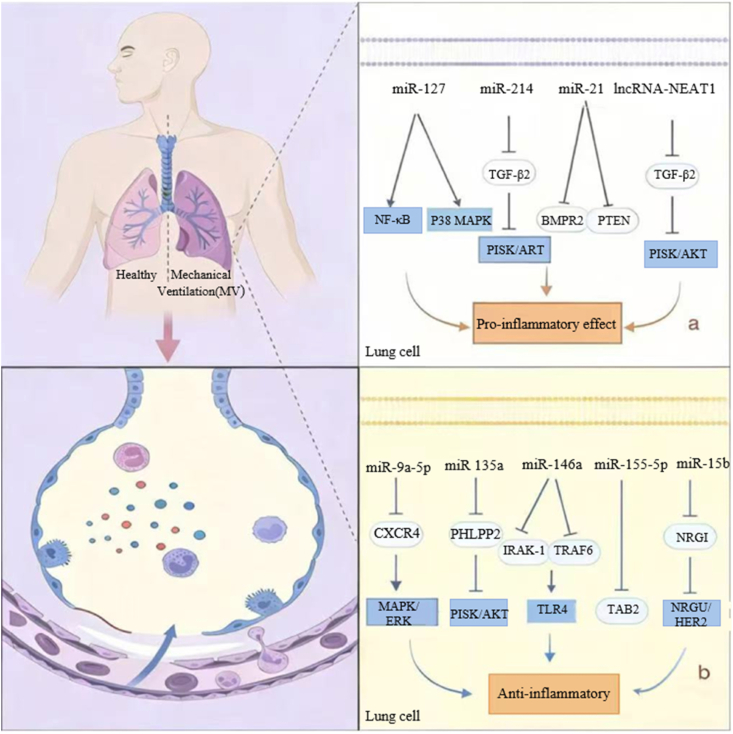
This diagram illustrates how ncRNAs participate in regulating the process of VILI biological injury. **a**: Regulation of pro-inflammatory ncRNAS; MiR-127 activates the NF-κB and p38 MAPK signaling pathways; MiR-214 targets and inhibits FGFR1, FGFR1 inhibits the PI3K/Akt pathway; Mir-21 targets and inhibits BMPR2 and PTEN; lncRNA targets and inhibits miR-21, and miR-21 activates the STAT3 pathway, thereby, promoting the VILI; **b**: miR-9a-5p targets and inhibits CXCR4, CXCR4 activates MAPK/ERK pathway; miR-135a targets and inhibits PHLPP2, PHLPP2 inhibits PI3K/AKT pathway; miR-146a targets and inhibits IRAK-1 and TRAF6 to activates TLR4 pathway; miR-155–5p targets and inhibits TAB2; miR-15b targets and inhibits NRG1,NRG1 inhibits NRG1/HER2 pathway, thereby Inhibit VILI inflammation.

Recent findings suggest that specific cells within lung tissue possess the ability to detect mechanical stimuli induced by lung hypertonicity. These cells transduce such mechanical signals into biochemical responses via particular signaling pathways [[38], [39], [40]]. This process activates inflammatory cells within the lung, thereby amplifying the inflammatory response. Consequently, there is a significant release of cytokines and inflammatory mediators, contributing to biotrauma [41]. The ncRNAs play a significant role in the pathogenesis of VILI by modulating inflammatory responses and influencing changes in vascular permeability (Fig. 2). A list of some ncRNAs recently reported to be involved in VILI is shown in Table 2.

**Table 2:** Key ncRNAs and their signaling pathways regulating inflammation and pulmonary edema in ventilator-induced lung injury (VILI).

	ncRNAs	Expression	Target	Pathway	References
**Promoting inflammation and pulmonary edema**					
	miR-127	↑	NA	NF-κB and p38 MAPK	[42]
	miR-214	↑	FGFR1	PI3K/AKT	[43]
	miR-21	↑	BMPR2 and PTEN	NA	[44]
	lncRNA NEAT1	↑	miR-20b	miR-20b/STAT3	[45]
	lncRNA Map2k3os	↑	NA	NA	[46]
**Suppressing inflammatory response**					
	miR-9a-5p	↓	CXCR4	MAPK/ERK	[47]
	miR135a	↓	PHLPP2	PI3k/Akt	[48]
	miR-146a	↑	IRAK-1 and TRAF6	TLR4 signaling	[11,49]
	miR-155–5p	↓	TAB2	NA	[50]
	miR-15b	↓	NRG1	NRG1/HER2	[51]

Abbreviation: NF-κB: nuclear factor kappa-B; MAPK: mitogen-activated protein kinase; FGFR1: fibroblast growth factor receptor 1; PI3K/AKT: phosphatidylinositol-3-kinase/protein kinase B; BMPR2: bone morphogenetic protein receptor type 2; PTEN: phosphatase and tension homolog; STAT3: signal transducer and activator of transcription 3; CXCR:C-X-C motif chemokine receptor; ERK: extracellular signal receptor-activated kinase; PHLPP2: PH Domain And Leucine Rich Repeat Protein Phosphatase 2; IRAK-1: Interleukin 1 Receptor Associated Kinase-1; TRAF6: Tumor Necrosis Factor-Associated Receptor 6; TLR4: Toll-like receptor 4; TAB2: TGF-beta activated kinase 1 (MAP3K7) binding protein 2; NRG1: Neuregulin 1; HER2: human epidermal growth factor receptor 2; NA: not available.

### 3.1 NcRNAs initiate and promote VILI

#### 3.1.1 MiRNA promotes oxidative stress and pro-inflammatory factor levels

Elevated levels of myeloperoxidase (MPO) and the secretion of pro-inflammatory mediators are key mechanisms underlying biological injury in VILI [[52], [53], [54], [55]]. NcRNAs can influence the inflammatory response by regulating both of these pathways. Cyclic mechanical stretching leads to lung damage by generating reactive oxygen species (ROS) in AECs. This process elevates oxidants like MPO, resulting in a harmful surge of reactive oxygen radicals [40,55]. Specifically, MiR-214 targets and inhibits fibroblast growth factor receptor 1 (FGFR1). At the same time, FGFR1 can inhibit its downstream pro-inflammatory phosphoinositide 3-kinase/protein kinase B (PI3K/Akt) pathway. Ultimately, this increases MPO activity and the levels of pro-inflammatory factors [43]. Therefore miR-214 enhances the PI3K/AKT signaling pathway through modulation of FGFR1 activity, thereby facilitating inflammatory processes [43]. MiR-127 promotes the inflammatory process in VILI by activating signaling pathways such as NF-κB and p38 MAPK, thereby disrupting the integrity of the alveolar capillary barrier. [42]. During VILI, in addition to the disruption of the alveolar capillary barrier, the integrity of the alveolar barrier is also compromised [44]. Studies have found that bone morphogenetic protein receptor type 2 (BMPR2) and phosphatase and tensin homolog (PTEN) play crucial roles in maintaining the integrity of the alveolar barrier. miR-21 can promote the initiation of inflammatory responses and lung edema associated with alveolar barrier disruption by downregulating the expression of BMPR2 and PTEN [44].

#### 3.1.2 LncRNA promotes pro-inflammatory factor levels

lncRNAs also have a significant impact on the regulation of proinflammatory mediators in VILI. Transfection of small interfering RNA (siRNA) of lnc mitogen-activated protein kinase 3, opposite strand (Map2k3os) can inhibit the expression of inflammatory cytokines and attenuate the inflammatory response. However, the precise molecular pathway underlying these effects remains undefined [46].

#### 3.1.3 Interdependence between lncRNA and MiRNA

However, in the complex pathophysiological network of VILI, ncRNAs do not act independently; they often form regulatory pathways through specific interaction patterns, jointly influencing the occurrence and progression of the disease.

Relevant experimental studies have confirmed that during VILI, there is a clear interactive regulatory relationship between long-chain non-coding RNA nuclear paraspeckle assembly transcript 1 (lncRNA NEAT1) and miR-20b in alveolar macrophages [45]. Specifically, lncRNA NEAT1 can accelerate the conversion of alveolar macrophages from the M1 type to the M2 type; meanwhile, this lncRNA can significantly upregulate the expression levels of inflammation-related factors such as interleukin-1β (IL-1β), interleukin-6 (IL-6), tumor necrosis factor-α (TNF-α), and inducible nitric oxide synthase (iNOS), thereby exacerbating pulmonary inflammatory responses [45]. To further elucidate its regulatory mechanism, bioinformatics analysis has shown that lncRNA NEAT1 can act as an endogenous competing RNA (ceRNA), directly binding to and inhibiting the activity of miR-20b through sequence complementarity. This, in turn, relieves its post-transcriptional inhibitory effect on the downstream target gene signal transducer and activator of transcription 3 (STAT3). Ultimately, by activating the STAT3 signaling pathway, it mediates the aforementioned pro-inflammatory effects and alveolar macrophage phenotype conversion [45].

The above studies indicate that ncRNAs play a key role in the inflammatory response of VILI, and their regulatory mechanisms provide potential directions for the treatment of mechanically ventilated patients. However, current research largely relies on mouse lung models, which, while useful for preliminary mechanism exploration, are difficult to fully translate to human pathology. Therefore, there is an urgent need to study human-derived lung cell models to verify the mechanistic role of ncRNAs in VILI.

### 3.2 The suppressive ncRNAs in VILI

#### 3.2.1 NcRNA inhibits oxidative stress and levels of inflammatory cytokines

Researchers found that the expression of miR-9a-5p was lower in VILI mice, while the expression of C-X-C motif chemokine receptor 4 (CXCR4) was higher. After overexpression of miR-9a-5p, the expression of inflammatory cytokines and MPO activity in lung tissues also decreased, and the degree of pathological damage was reduced [47]. Additional research demonstrated that the suppressive influence of miR-9a-5p was due to a reduction in CXCR4 expression. This downregulation resulted in the subsequent inactivation of the MAPK/extracellular signal receptor-activated kinase (ERK) signaling cascade [47]. In a model of VILI in human umbilical vein endothelial cells (HUVEC), overexpression of miR-135a led to reductions in apoptosis, barrier dysfunction, reactive oxygen species (ROS), and cytokine levels. This was associated with the activation of the PI3K/Akt signaling pathway. MiR-135a potentially exerts its effects by inhibiting the PH Domain And Leucine Rich Repeat Protein Phosphatase 2 (PHLPP2). The observed decrease in PHLPP2 expression, alongside the activation of the PI3K/Akt pathway, supports the inhibitory role of miR-135a [48].

#### 3.2.2 NcRNA inhibits key proteins in the signaling pathway

NcRNAs can suppress inflammatory responses through alternative signaling pathways. The Toll-like receptor 4 (TLR4) pathway is known to promote inflammation in VILI. [56]. MiR-146a modulates inflammatory responses via a negative feedback loop involving Interleukin 1 Receptor Associated Kinase-1 (IRAK-1) and Tumor Necrosis Factor-Associated Receptor 6 (TRAF6), thereby regulating TLR and inflammatory signaling pathways [11,49]. Another study indicated that overexpression of miR-155–5p diminishes inflammatory cell infiltration and alleviates lung injury by negatively regulating TGF-beta activated kinase 1 (MAP3K7) binding protein 2 (TAB2) [50]. Furthermore, miR-15b reduces alveolar epithelial permeability by downregulating Neuregulin 1 (NRG1), decreasing its expression in cyclically stretched alveolar epithelial cells (AECs), and inhibiting the activation of human epidermal growth factor receptor 2 (HER2) [51]. Collectively, these findings highlight the pivotal regulatory functions of inhibitory ncRNAs in the development of VILI. Nonetheless, the existing evidence is limited, emphasizing the need for additional mechanistic and translational research.

## 4 NcRNAs participate in VILI via regulating mechanotransduction

In addition to the release of pro-inflammatory mediators and the production of reactive oxygen species (ROS), the mechanism leading to VILI biological injury also involves mechanical signal transduction as an important link. Mechanical transduction is crucial in the process of converting mechanical stimuli into biochemical signals during ventilation, and ncRNAs are also closely related to this process.

### 4.1 NcRNAs in intracellular mechanotransduction

**Fig. 3:**
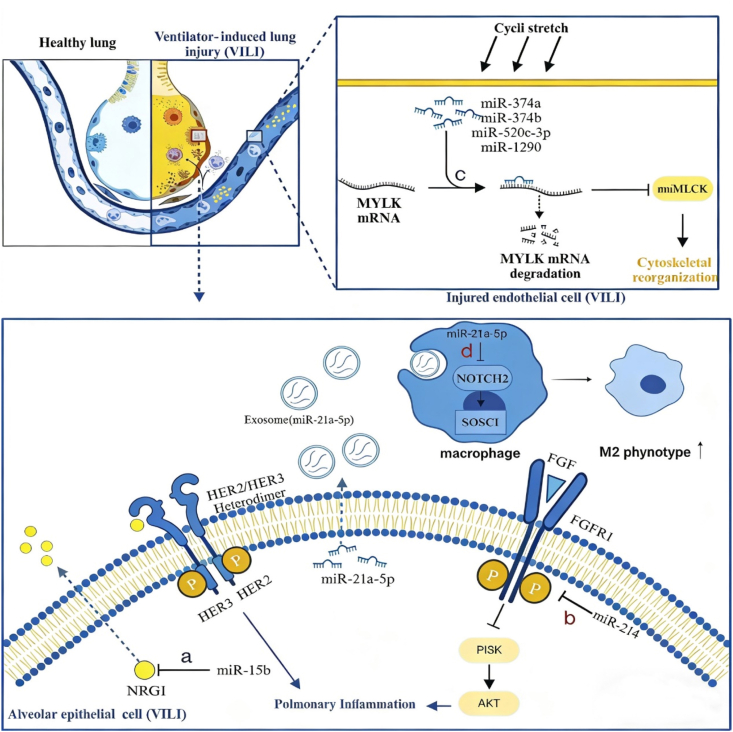
NcRNAs participate in mechanical signal transduction both intercellularly and extracellularly. **a**: MiR-15b suppresses the HER ligand NRG1, thereby inhibiting HER2 activation in lung tissue cells; **b**: MiR-214 directly suppresses FGFR1 while augmenting PI3K/Akt activity; **c**: MiR-374a/b, miR-520c-3p, and miR-1290 suppress the expression of MYLK, thereby reducing the levels of nMLCK in pulmonary endothelial cells and inhibiting cytoskeletal reorganization; **d**: Exosomal miR-21a-5p from AECs drives M2 macrophage polarization by inhibiting the Notch2/SOCS2 axis, mediating anti-inflammatory effects.

Mechanotransduction is crucial for transforming mechanical stimuli into biochemical signals during the ventilation process. Prior studies on mechanical signal transduction have demonstrated that the activation of protein kinases serves as a fundamental mechanism in this conversion [57,58]. Certain studies have explored the regulatory mechanisms governing protein kinase activity in VILI mediated by ncRNAs. These mechanisms involve receptor protein mechanosensors located at the cell membrane, the cytoskeleton, and intracellular signaling components. Research findings suggest that HER2 and FGFR, which are classified as receptor tyrosine kinases due to their molecular structure, can serve as sensors at the cell membrane for detecting mechanical stimuli [59,60]. In lung tissue cells, MiR-15b functioned as a suppressor of HER ligand NRG1 (Fig. 3a), which consequently hinders the activation of HER2 [51]. In contrast to the aforementioned finding, it was observed that miR-214 suppresses the expression of its target gene FGFR1, augments the activity of the PI3K/AKT pathway (Fig. 3b) and consequently demonstrates a pro-inflammatory effect [43].

Similarly, these conclusions still need to be further validated using human lung cell models.

The cytoskeleton functions as a dynamic and adaptable framework within cells. It reorganizes in response to mechanical stimuli and facilitates signal transmission. This process is governed by biomechanical models that consider the tension present in different lung tissue regions [61]. The main structures of cytoskeleton include actin filaments, intermediate filaments, and microtubules, and are acted on by three families of motor proteins: myosin, kinesin, and dynein [62]. The activation of the non-muscle isoform of myosin light chain kinase (nmMLCK) has been shown to facilitate myosin light chain phosphorylation [63] and enhance actin cytoskeletal reorganization [64,65] and govern the transportation of inflammatory cells [66].

A study involving human lung endothelial cells demonstrated that the expression levels of miR-374a, miR-374b, miR-520c-3p, and miR-1290, regulating the myosin light chain kinase (MYLK) (the gene encoding the nmMLCK multifunctional enzyme), exhibited a decrease (Fig. 3c) [63]. Conversely, the levels of nmMLCK showed a rise [63]. After reverse intervention using miRNA mimics to transfect lung endothelial cells, nmMLCK expression decreased, attenuating the high permeability of endothelial cells during lung injury. The aforementioned observation implies that ncRNAs could potentially play a role in modulating the transmission mechanism of mechanical signaling through their influence on the cytoskeleton's motor reorganization function.

The nmMLCK gene, which serves as a barrier-regulated gene, has been identified as a viable molecular target of acute lung injury (ALI) [64]. The suppression of nmMLCK by miR-374a, miR-374b, miR-520c-3p, and miR-1290 highlights their therapeutic potential for ameliorating vascular permeability and edema in ALI and VILI [63]. This technique might reduce the risk and severity of ALI and VILI.

### 4.2 NcRNAs in extracellular exosomes

Under normal physiological conditions, the maintenance of lung homeostasis is significantly influenced by exosome-mediated intercellular communication between AECs and macrophages [67,68]. The synchronization of cellular responses in lung tissues necessitates the transfer of mechanical force signals across cells. Exosomes play a significant role in facilitating intercellular communication of mechanical force signals by modulating the phenotypes of recipient cells by targeting mRNA molecules.

The investigators discovered that miRNA-21a-5p in exosomes derived from AECs promotes M2 macrophage polarization facilitating the anti-inflammatory effects of macrophages via inhibition of the Notch2/suppressor of cytokine signaling 1 (Notch2/SOCS1) axis (Fig. 3d) [69,70]. Furthermore, it has been proven that adipose-derived exosomes can mitigate the detrimental effects of mechanical stretch on endothelial barrier integrity and inflammatory response in the pulmonary vasculature. However, the specific mechanism by which exosomal miRNAs mediate this protective effect remains unknown [71]. The above findings indicate that exosomes serve as a significant mechanism for modulating intercellular and interorgan communications in VILI, with ncRNAs within exosomes potentially assuming a crucial function in this process.

Mechanical signal transduction in VILI is a complex process that is influenced by ncRNAs. Initially, mechanical signals are detected by sensors on the cell membrane, such as HER2 and FGFR mentioned above, which can act as cell membrane sensors to detect mechanical stimuli [62,63]. The mechanically sensitive ion channel Piezo1 can sense mechanical ventilation stimuli in acute respiratory distress syndrome (ARDS). Once activated, mediates the conversion of extracellular mechanical signals into intracellular molecular signaling cascades. However, it remains unclear whether it performs a similar cell membrane sensor function in ventilator-induced lung injury (VILI). Furthermore, whether its expression and activity are regulated by ncRNAs requires further in-depth investigation [72]. Subsequently, triggering intracellular biochemical signaling responses. Convert the physical forces sensed by the receptors into biochemical responses [73]. Ultimately, these signals promote the activation of cellular inflammatory responses through the cytoskeleton and intracellular signaling pathways [[63], [64], [65], [66]]. In addition, neighboring cells in lung tissue can also be affected through extracellular signaling mechanisms, such as the release of exosomes [69,70]. Therefore, ncRNAs play a key role in the mechanical signal transduction of VILI, serving as an important regulatory mechanism.

## 5 Discussion

### 5.1 Reactivation of lung development pathways

During lung development, TGF-β2 significantly inhibits lung epithelial cell differentiation/maturation and downregulates pulmonary surfactant synthesis/secretion [16,27]. Existing Chinese studies confirm abnormal activation of the TGF-β2 in VILI [74]. Periodic lung tissue stretching induces excessive reactive oxygen species (ROS) production via oxidative stress [40,55], and ROS further upregulate TGF-β2 [74]. Elevated TGF-β2 exacerbates alveolar and interstitial edema in VILI by impairing endothelial barrier function [75,76].

In addition, the Wnt signaling pathway, which promotes alveolar cell differentiation and the production of alveolar surfactant, can also be reactivated. [77,78]. VILI significantly increases Wnt5a protein expression in lung tissue [77,79]; it specifically activates the non-canonical Wnt signaling pathway [80]to upregulate Wnt-induced secreted protein 1 (WISP1), which exacerbates inflammatory cell infiltration, pulmonary edema, and alveolar septal thickening, thereby contributing to VILI-related pathological damage [77]. In contrast, the canonical Wnt/β-catenin pathway is downregulated post-mechanical ventilation. Its activation reduces inflammation and apoptosis, mitigates lung injury, and thus exerts a protective effect against VILI [78].

The above studies indicate that Key molecules in lung development pathways exhibit differential expression during the progression of VILI according to their different roles in the development process. It suggests that their potential value as diagnostic biomarkers for VILI. In addition, modulating developmental pathways (such as the Wnt pathway) can balance repair and inflammatory responses, providing new therapeutic targets and supplementary directions for existing treatment approaches. But the molecular mechanisms of VILI are very complex. The 'interactions' of different developmental pathways in VILI have not yet been clarified. Moreover, most studies are still limited to animal experiments, and there is a lack of clinical research to verify their safety and effectiveness. This also offers guidance for future research.

### 5.2 Prediction of ncRNAs as a diagnostic biomarker for VILI

NcRNAs are highly promising biomarkers for VILI diagnosis, commonly detected in bronchoalveolar lavage fluid, serum, and lung tissue. At 0.2-Hz, 20 cmH_2_O oscillatory pressure, mechanosensitive miR-146a activates the NF-κB inflammatory pathway within 1–4 h [11]. mechanosensitive miRNAs generally responding early in mechanotransduction to assist inflammatory signal activation. Additionally, anti-inflammatory miR-146a has shown initial success in VILI therapeutic studies [81]. Studies found that in vivo overexpression of miR-146a via untargeted lipid nanoparticles effectively reduces cytokine release and barrier disruption post-mechanical stretching, but its clinical applicability is limited by the need for a high initial dose [82]. Another study used mannosylated lipid nanoparticles (specifically targeting macrophages) for preferential miR-146a delivery, demonstrating that even lower doses effectively alleviate lung inflammation—suggesting mannose-modified lipid nanoparticles hold therapeutic potential for mitigating VILI-related lung injury [83].

However, the safety of these therapeutic measures remains to be investigated. 1. Off-target effect: The mannose receptor is expressed on hepatic endothelial cells and dendritic cells (DCs) [84]. Future studies should focus on tissue-specific targeting to avoid damaging non-target cells. 2. Delivery system safety: The above therapies depend on lipid nanoparticles (LNPs) for lung-targeted delivery. It is critical to evaluate LNP biocompatibility and control dosage to prevent lung epithelial/endothelial cytotoxicity, which may impair gas exchange.

During lung development, the miR-29 family promotes the differentiation of AEC II and the production of pulmonary surfactants by suppressing TGF-β2 signaling [16]. Meanwhile, several studies have identified miR-29 as a potential biomarker and ideal therapeutic target for lung diseases including bronchopulmonary dysplasia (BPD), idiopathic pulmonary fibrosis (IPF), and non-small cell lung cancer (NSCLC) [[85], [86], [87]]. In terms of VILI, Vaporidi et al. used bioinformatics technology to predict a VILI-associated miRNA gene network based on the TGF-β signaling pathway, with SMAD4 as the central node [44]. Additionally, bioinformatics analysis has predicted the miR-29 family to act as a key gene in VILI [9]. Considering the role of miR-29 in both physiological and pathological processes of the lung, we hypothesize that the miR-29 family could serve as a potential biomarker for VILI. This proposal may provide novel insights into the diagnostic strategies for VILI, though further validation studies are required to confirm this potential.

Consequently, identifying additional mechanosensitive microRNAs as potential biomarkers or therapeutic targets could enhance early detection of ventilator-induced lung injury (VILI). This advancement would facilitate timely and targeted interventions, potentially improving patient outcomes.

### 5.3 Possible future research directions

#### 5.3.1 Circ RNAs in VILI

Circular RNAs (circ RNAs) are covalently closed, single-stranded ncRNAs that regulate key physiological and pathological processes (transcription, translation, immune responses, oncogenesis, etc.) [88,89]. Research on circ RNAs (beyond miRNAs and lnc RNAs) in VILI is limited. Recent high-throughput sequencing of lung tissues from VILI-induced mice identified 171 upregulated and 114 downregulated circ RNAs, linking circ RNA expression changes to VILI progression [90]. In circUBR1 knockdown models, lung injury was attenuated by modulating the miR-20a-5p/GGPPS1 pathway [91].

Circ RNA can engage in the pathological mechanisms of pulmonary inflammation through its role as a competitive endogenous RNA (ce RNA) by binding microRNAs (miRNAs). In a model of sepsis-induced acute lung injury, circVMA21 in human normal lung epithelial cells mitigated lung damage in septic rats by modulating the miR-497–5p/CD2AP signaling pathway [92]. In a mouse lung microvascular endothelial cell (MPVECs), circ3P1 reduced pro-inflammatory cytokine production and apoptosis in ALI by regulating miR-21 [93]. Conversely, circKlhl2 accelerated sepsis-induced ALI by regulating the miR-29b-3p/ROCK1 axis [94].

The precise mechanisms by which circ RNAs influence VILI remain largely undefined, however, it is evident that circ RNAs potentially play a significant role in mediating inflammatory processes associated with VILI.

Most current related studies primarily focus on neonatal models. Research on the regulatory mechanisms of ncRNAs in adult VILI remains scarce. Therefore, the existing conclusions about the regulatory roles of ncRNAs in lung development and VILI, as well as their scientific inference as potential therapeutic targets, have significant limitations in applicability and generalizability to adult populations. In the future, research should focus on constructing adult-specific models to deeply analyze the core regulatory pathways and molecular interaction networks of ncRNAs in the onset and progression of adult VILI, providing targeted theoretical support for precise interventions in adult VILI.

## 6 Conclusion

The management of VILI still faces major obstacles; despite progress, challenges (from clarifying molecular pathogenesis to developing targeted therapies) remain, requiring multidisciplinary efforts. Since ncRNAs regulate nearly all physiological and pathological processes, studying their link to VILI is essential. This paper introduces ncRNAs’ (mainly miRNAs and lncRNAs) regulatory roles in pulmonary physiology and VILI, focuses on the molecular mechanism of biologic injury, and details their regulatory targets and related pathways—providing new insights for VILI treatment. Emerging evidence shows ncRNAs are key regulators of VILI-associated mechanotransduction, with potential as diagnostic biomarkers or therapeutic targets to improve clinical outcomes. However, current scientific-technological limitations and clinical resource constraints prevent their immediate application in VILI treatment or prognosis. Future research needs to be more comprehensive and in-depth to find more effective VILI therapies.

## 7 Search strategy

### 7.1 Database

A comprehensive literature search was performed exclusively in PubMed (https://pubmed.ncbi.nlm.nih.gov/), the most authoritative biomedical database, to identify all relevant studies published up to the retrieval date.

### 7.2 Key words

The search strategy combined subject headings (MeSH terms) and free-text keywords related to “non-coding RNA” and “ventilator-induced lung injury”, with logical operators (“AND”) to expand the retrieval scope.

### 7.3 Category keywords/MeSH terms

“Ventilator-Induced Lung Injury"[Mesh] OR “ventilator induced lung injury"[tiab] OR “ventilator-associated lung injury"[tiab] OR VILI[tiab] OR “mechanical ventilation lung injury"[tiab]

“RNA, Untranslated"[Mesh] OR “Untranslated RNA"[tiab] OR “non-coding RNA"[tiab] OR “noncoding RNA"[tiab] OR ncRNA[tiab] OR “Long Noncoding RNA"[tiab] OR lncRNA[tiab] OR “MicroRNAs"[Mesh] OR microRNA[tiab] OR miRNA[tiab] OR “Circular RNA"[tiab] OR circRNA[tiab] OR “PIWI-Interacting RNA"[tiab] OR piRNA[tiab].

### 7.4 Inclusion criteria

1. Study type: Original research (in vitro cell experiments, in vivo animal models, or clinical studies), reviews, excluding meta-analyses, case reports, editorials, and conference abstracts.
2. Research content: Studies investigating the regulatory roles of non-coding RNAs (lncRNA, miRNA, etc.) in ventilator-induced lung injury (VILI), including mechanisms such as inflammation, cell apoptosis, macrophage polarization, or epithelial-mesenchymal transition.
3. Outcome indicators: Studies reporting differential expression of non-coding RNAs in VILI models (vs. control groups) or verifying their functional effects via overexpression/knockdown.
4. Language: English-only publications (to avoid translation bias).
5. Data availability: Studies with complete raw data or clear result descriptions.

### 7.5 Exclusion criteria

1. Studies not focusing on VILI (e.g., lung injury induced by sepsis, trauma, or toxins alone).
2. Studies where non-coding RNA is not the core regulatory molecule (e.g., only mentioned as a secondary finding).
3. Duplicate publications (the latest or most comprehensive study was retained).
4. Incomplete data, unreadable full text, or non-original research.
5. Studies using non-standard VILI models (e.g., ventilation parameters not clearly defined).

**Figure:**
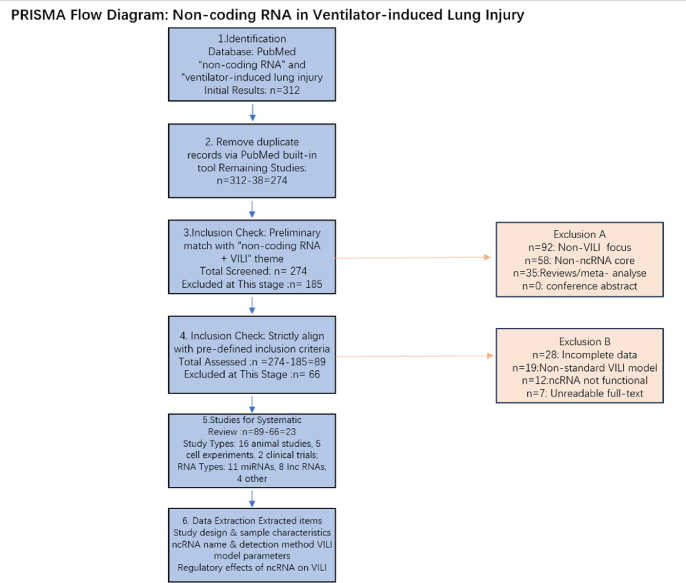

## CRediT authorship contribution statement

**Qianqi Xiong:** Writing – original draft. **Ziyang Zhang:** Writing – original draft. **Yiping Bai:** Writing – review & editing, Supervision. **Li Liu:** Writing – review & editing, Supervision. **Yue Zhang:** Writing – review & editing, Supervision. **Jing Jia:** Writing – review & editing, Supervision. **Yingying Zhang:** Writing – review & editing, Supervision, Funding acquisition.

## Funding

This study is supported by Sichuan Science and Technology Program (2022YFS0632), the affiliated hospital of 10.13039/501100014895Southwest Medical University (24233), and National College Students' Innovation and Entrepreneurship Fund (Ziyang Zhang, 202310632070), National College Students' Innovation and Entrepreneurship Fund (Qianqi Xiong, 202310632114). Study funders were involved in soliciting project specifications and regularly reviewing the progress of projects.

## Declaration of competing interest

The authors declare that they have no known competing financial interests or personal relationships that could have appeared to influence the work reported in this paper.
